# β-lactam antibiotic concentrations during continuous renal replacement therapy

**DOI:** 10.1186/cc13886

**Published:** 2014-05-22

**Authors:** Marjorie Beumier, Giuseppe Stefano Casu, Maya Hites, Lucie Seyler, Frederic Cotton, Jean-Louis Vincent, Frédérique Jacobs, Fabio Silvio Taccone

**Affiliations:** 1 Department of Intensive Care, Erasme Hospital, Université Libre de Bruxelles (ULB), Route de Lennik, 808, 1070 Brussels, Belgium; 2 Department of Infectious Diseases, Erasme Hospital, Université Libre de Bruxelles (ULB), Route de Lennik, 808, 1070 Brussels, Belgium; 3 Department of Clinical Biochemistry, Erasme Hospital, Université Libre de Bruxelles (ULB), Route de Lennik, 808, 1070 Brussels, Belgium

## Abstract

**Introduction:**

The use of standard doses of β-lactam antibiotics during continuous renal replacement therapy (CRRT) may result in inadequate serum concentrations. The aim of this study was to evaluate the adequacy of unadjusted drug regimens (i.e., similar to those used in patients with normal renal function) in patients treated with CRRT and the influence of CRRT intensity on drug clearance.

**Methods:**

We reviewed data from 50 consecutive adult patients admitted to our Department of Intensive Care in whom routine therapeutic drug monitoring (TDM) of broad-spectrum β-lactam antibiotics (ceftazidime or cefepime, CEF; piperacillin/tazobactam; TZP; meropenem, MEM) was performed using unadjusted β-lactam antibiotics regimens (CEF = 2 g q8h; TZP = 4 g q6h; MEM = 1 g q8h). Serum drug concentrations were measured twice during the elimination phase by high-performance liquid chromatography (HPLC-UV). We considered therapy was adequate when serum drug concentrations were between 4 and 8 times the minimal inhibitory concentration (MIC) of *Pseudomonas aeruginosa* during optimal periods of time for each drug (≥70% for CEF; ≥ 50% for TZP; ≥ 40% for MEM). Therapy was considered as early (ET) or late (LT) phase if TDM was performed within 48 hours of antibiotic initiation or later on, respectively.

**Results:**

We collected 73 serum samples from 50 patients (age 58 ± 13 years; Acute Physiology and Chronic Health Evaluation II (APACHE II) score on admission 21 (17–25)), 35 during ET and 38 during LT. Drug concentrations were above 4 times the MIC in 63 (90%), but above 8 times the MIC in 39 (53%) samples. The proportions of patients with adequate drug concentrations during ET and LT were quite similar. We found a weak but significant correlation between β-lactam antibiotics clearance and CRRT intensity.

**Conclusions:**

In septic patients undergoing CRRT, doses of β-lactam antibiotics similar to those given to patients with normal renal function achieved drug levels above the target threshold in 90% of samples. Nevertheless, 53% of samples were associated with very high drug levels and daily drug regimens may need to be adapted accordingly.

## Introduction

Sepsis is a major cause of morbidity and mortality in critically ill patients [1,2]. Early and adequate antibiotic therapy in this population is crucial to maximize chances of survival [3-6]. β-lactam antibiotics are widely used as first-line therapy in septic patients and are particularly effective against bacteria less susceptible to other antibiotics, such as *Pseudomonas aeruginosa*. These drugs have a time-dependent antibacterial activity and the best pharmacodynamic parameter to predict their efficacy is the time during which their serum concentration is above the minimal inhibitory concentration (MIC) of the pathogen [7].

Sepsis may significantly alter antibiotic pharmacokinetics (PK). In particular, volume of distribution (Vd) may increase because of fluid resuscitation and capillary leakage, whereas increased cardiac output may promote augmented renal clearance and drug elimination [7]. Thus, insufficient antibiotic concentrations may occur in septic patients receiving standard antibiotic doses, potentially leading to therapeutic failure and encouraging development of antimicrobial resistance [7,8]. However, antibiotic PK can vary considerably during critical illness; for example, acute kidney injury (AKI), which commonly complicates sepsis, alters antibiotic elimination, leading to drug accumulation [9]. The use of continuous renal replacement therapy (CRRT) may further alter antibiotic PK. Nevertheless, current recommendations on antibiotic dosing during CRRT [10] are based on studies that included a limited number of patients, with varying inclusion/exclusion criteria and who received different types of RRT [11-13]. Indeed, Roberts *et al*. showed a great variability in β-lactam antibiotics concentrations in critically ill patients treated with CRRT [14]. Moreover, in a prospective study, Seyler *et al*. showed that the recommended doses for broad-spectrum β-lactam antibiotics were largely insufficient to maintain therapeutic serum concentrations for the treatment of *P. aeruginosa* in septic patients [15]. The authors suggested the use of β-lactam antibiotics doses similar to those used in patients without renal failure, at least during the first days of treatment in this population. The aim of the present study was, therefore, to evaluate the adequacy of this dosage strategy in septic patients treated with CRRT and to evaluate the influence of CRRT intensity on drug clearance.

## Patients and methods

### Study design and inclusion criteria

Since December 2009, patients undergoing CRRT in our unit, who require treatment with β-lactam antibiotics receive doses similar to those used in patients with normal renal function. We therefore reviewed data from all adult patients admitted to the 35-bed Department of Intensive Care of Erasme University Hospital, Brussels between January 2010 and November 2011. Inclusion criteria were: a) diagnosis of severe sepsis or septic shock according to standard criteria [16]; b) therapy with broad-spectrum β-lactam antibiotics (ceftazidime or cefepime (CEF), piperacillin/tazobactam (TZP) or meropenem (MEM)), given at the usual doses (CEF = 2 g q8h; TZP = 4 g q6h; MEM = 1 g q8h); c) AKI treated with CRRT; d) residual creatinine clearance (CrCl) <30 mL/minute; e) at least one therapeutic drug monitoring (TDM) sample taken during the CRRT treatment. Exclusion criteria were burns, cystic fibrosis and the use of extracorporeal membrane oxygenation (ECMO) therapy. The Ethics Committee of Erasme Hospital approved the study protocol waiving the need for informed consent in view of its retrospective nature.

### Indications for therapeutic drug monitoring

The choice of antibiotic was made by the attending physician. Since October 2009, TDM of broad-spectrum β-lactam antibiotics has been routinely performed in our department, especially in patients with severe sepsis or septic shock, overweight patients, patients with multidrug-resistant strains of infection, transplanted patients or patients receiving extracorporeal therapies. All patients undergoing TDM are included in a prospective database, in which drug regimens, date and hours of drug sampling are recorded for each sample.

### Data collection

We recorded the demographics, comorbidities, admission diagnosis, biological and microbiological (site of infection and pathogens), length of ICU and hospital stay and overall mortality for all patients. The severity of illness was assessed by the acute physiology and chronic health evaluation (APACHE) II score [17] on admission and the sequential organ failure assessment (SOFA) score [18] was recorded on the day of TDM. Treatment with vasopressors or mechanical ventilation was also recorded. Characteristics of CRRT, including dialysate rate, ultrafiltrate rate, and blood flow were recorded. CRRT intensity (mL/kg/h) was calculated using the following formula:

$$\left(\text{Dialysate}\;\text{rate}\;\left(\text{mL}/h\right)\;+\;\text{Ultrafiltrate}\;\left(\text{mL}/h\right)\right)/\text{Weight}\;\left(\text{kg}\right).$$

Residual creatinine clearance (CrCl, mL/minute) was calculated from urine using the formula:

$$\begin{array}{ll} \;\left(\text{Urine}\;\text{output},\;\text{mL}\right) & *\left(\text{Urinary}\;\text{creatinine}\;\text{concentration},\text{mg}/\text{dL}\right)\\ & /\left(\text{Serum}\;\text{creatinine}\;\text{concentration},\text{mg}/\text{dL}\right)\\ & *\left(\text{Time}\;\text{of}\;\text{urine}\;\text{collection},\;\text{minutes}\right). \end{array}$$

### Pharmacokinetics analyses

β-lactam antibiotics concentrations were determined on two blood samples (3 mL) drawn during the antibiotic elimination phase: one 2 h (T2) after a 30-minute drug infusion and the other just before the next dose administration (T0). The drug was administered over a 30-minute period by using an infusion pump, and the tubing was flushed with 0.9% sodium chloride after the dose was administered. Nurses noted the exact sampling time in the ICU patient data monitoring system (PDMS, Picis Critical Care Manager, Picis Inc., Wakefield, USA). Samples were immediately put on ice and sent to the clinical chemistry laboratory, where they were centrifuged at 3000 rpm at 4°C for 10 minutes; the supernatant was then removed and analyzed using high-performance liquid chromatography connected to UV spectro-photometry (HPLC-UV), as previously reported [19]. For TZP, only piperacillin levels were measured. A one-compartment model was chosen to perform PK analyses and, assuming a reached steady-state, T0 and T2 concentrations were used to determine Vd, total drug clearance (CL) and elimination half-life (t_1/2_) [15]. Importantly, TDM results were available to clinicians but dose adjustments were performed only after a multidisciplinary discussion. Early (ET) and late (LT) phases of therapy corresponded to drug levels assessed (TDM) within 48 h of start of antibiotics or later on, respectively.

### Clinical breakpoints and adequacy of therapy

Adequacy of therapy was defined as drug concentrations reaching the minimal target of four times the MIC of *P. aeruginosa*; this parameter was expressed as the time in hours above four times the target MIC (T >4 × tMIC ) or the percentage of time above four times the target MIC (% T >4 × tMIC). We used the clinical breakpoints for *P. aeruginosa* as defined by the European Committee on Antimicrobial Susceptibility Testing (EUCAST): 8 μg/mL for CEF, 16 μg/mL for TZP and 2 μg/mL for MEM [20]. Thus, minimal target concentrations were 32 μg/mL, 64 μg/mL and 8 μg/mL for CEF, TZP and MEM, respectively. Because of specific drug properties, such as the post-antibiotic effect or post-antibiotic leukocyte enhancement effects, the optimal% T >4 × tMIC may differ between antibiotics: optimal periods of time were defined as ≥40%, ≥70% and ≥50% of the dosing interval for MEM, CEF and TPZ, respectively [15]. Under-dosing was thus defined as drug levels below minimal target concentrations for optimal periods of time. Excessive drug concentrations were arbitrarily defined as those exceeding eight times the target MIC for optimal periods of time. Finally, we calculated the proportion of TDMs with insufficient % T >4 × MIC for different MIC values.

### Continuous renal replacement therapy

CRRT was initiated according to local practice. The main indications for CRRT were: metabolic acidosis (pH <7.2); electrolyte disturbances (especially potassium levels exceeding 6 mEq/L); drug intoxication; fluid overload (that is, pulmonary edema) and blood urea levels >200 mg/dL. In our department CRRT is the standard of care, and standard hemodialysis or sustained low-efficiency dialysis are used only for hemodynamically stable patients before discharge to the floor. CRRT is performed through a double-lumen catheter inserted into the subclavian, femoral or internal jugular vein; continuous veno-venous hemodiafiltration (CVVHDF) or hemofiltration (CVVHF) were performed using standard equipment (Prisma or PrismaFlex, Gambro Hospal, Bologna, Italy), with a polyacrilonitrile cylinder (AN69 - Hospal, Meyzieu, France) or polysulfone (PS) hemofilters (Gambro Lundia AB, Lund, Sweden). Anticoagulation was obtained using either a systemic heparin infusion or a citrate infusion within the CRRT circuit. Initial blood flow was set at around 130 to 150 mL/minute and the ultrafiltrate rate was adjusted to provide at least 15 to 20 mL/kg/h. Dialysate was generally used during the first 24 to 48 h of therapy.

### Statistical analysis

Statistical analyses were performed using the SPSS 18.0 for Windows NT software package (SPSS Inc. 2004, Chicago, IL, USA). Descriptive statistics were performed for all study variables. Discrete variables are expressed as counts (percentage), and continuous variables as mean ± SD or median (25th to 75th percentiles). The Student *t*-test was used to assess differences between groups (early versus late). A *P*-value <0.05 was considered statistically significant.

## Results

Fifty patients met the inclusion criteria during the study period; drug regimens remained unchanged over the study period. A total of 73 TDMs were analyzed, 35 performed during ET and 38 during LT. ICU and hospital mortality rates were 50% and 60%, respectively. The median APACHE II score was 21 on admission (Table 1). Sepsis was mainly due to Gram-negative bacteria (GNB) (73%), including *P. aeruginosa* in 18 patients. The lung was the most frequent site of infection (n = 23) (Table 2). At the moment the study drug was initiated in each patient, CRRT had been ongoing for 2 (0 to 3) days.

**Table 1:** Characteristics of patients on ICU admission and ICU outcomes

	**Patients (n = 50)**
Age, years	59 (51 to 67)
Men/women, n	33/17
Body weight, kg	75 (68 to 85)
Body mass index, kg/m^2^	26.3 (23.9 to 27.9)
COPD/asthma, n (%)	8 (16)
Cardiopathy, n (%)	19 (38)
Diabetes mellitus, n (%)	17 (34)
sCr >2 mg/dL, n (%)	13 (26)
Liver cirrhosis, n (%)	18 (36)
Cancer, n (%)	7 (14)
Immunosuppressive agents, n (%)	20 (40)
Organ transplantation, n (%)	17 (30)
Medical admission, n (%)	39 (78)
APACHE II score on admission	21 (17 to 25)
Lactate levels on admission, mEq/L	2.6 (1.5 to 3.9)
Mechanical ventilation during ICU stay, n (%)	36 (72)
ICU stay, days	15 (10 to 21)
Hospital stay, days	23 (15 to 53)
ICU mortality, n (%)	25 (50)
Hospital mortality, n (%)	30 (60)

Data are presented as count (percentage) or median (25th to 75th percentiles). COPD, chronic obstructive pulmonary disease; APACHE, acute physiology and chronic health evaluation; sCr, serum creatinine before ICU admission.

**Table 2:** Characteristics of infections, identified Gram-negative pathogens and β-lactam antibioticss

	**Number of patients (n = 50)**
**Sites of infection**	
Lungs	23 (46)
Abdomen	15 (30)
Skin and soft tissues	3 (6)
Urinary tract	1 (2)
Other sites of infection	5 (10)
Positive blood cultures	6 (12)
**Pathogens**	
*Enterobacteriaceae*	17 (34)
*Pseudomonas aeruginosa*	18 (36)
*Acinetobacter baumannii*	3 (6)
**Antimicrobial treatment**	
Ceftazidime or cefepime	7 (14)
Piperacillin-tazobactam	16 (32)
Meropenem	32 (64)

Data are presented as count (percentage).

Thirty-two patients (64%) were treated with MEM, 16 (32%) with TPZ and 7 (14%) with CEF, with 5 patients receiving two different study drugs over the CRRT period. Drug CL was significantly lower in the LT phase compared to the ET phase for patients receiving CEF, despite similar CRRT intensity and SOFA score; this effect resulted in higher drug concentrations in the LT compared to the ET phase (Table 3); TZP concentrations were significantly higher in the LT compared to the ET phase, despite similar PK and CRRT parameters. Median concentrations and PK parameters were similar for MEM in the two treatment phases.

**Table 3:** Pharmacokinetic and pharmacodynamic data and characteristics of continuous renal replacement therapy (CRRT)

**Time from start of antibiotic to sampling, days**	**Early TDM (n = 35) 2 (1–2]**			**Late TDM (n = 38) 5 (3–14]**		
	**CEF (n = 3)**	**MEM (n = 25)**	**TZP (n = 7)**	**CEF (n = 6)**	**MEM (n = 19)**	**TZP (n = 13)**
**Volume of distribution, L**	19.4 (15.8 to 23.1)	33.3 (9.2 to 86.5)	18.5 (11.5 to 61.3)	19.2 (11.4 to 32.9)	28.8 (17.26 to 83.78)	18.2 (7.94 to 52.80)
**Volume of distribution, L/kg**	0.26 (0.24 to 0.28)	0.39 (0.11 to 1.20)	0.24 (0.17 to 0.87)	0.21 (0.16 to 0.51)	0.37 (0.23 to 1.18)	0.22 (0.11 to 0.76)
**t**_**1/2**_**, h**	4.3 (1.5 to 9.7)	3.9 (1.8 to 22.8)	3.0 (1.5 to 61.3)	8.0 (6.0 to 35.3)*	3.6 (1.8 to 10.7)	4.8 (1.8 to 7.8)
**Drug clearance, mL/h**	52.4 (27.3 to 120.9)	72.3 (21.4 to 283.0)	54.0 (47.3 to 185.6)	23.3 (5.4 to 58.5)*	98.3 (21.6 to 174.1)	51.0 (16.1 to 140.83)
**Concentration at T0, mg/L**	28.2 (26.1 to 49.0)	7.7 (2.0 to 32.2)	63.2 (15.3 to 115.2)	64.3 (3.2 to 139.0)*	6.8 (2.7 to 23.2)	80.0 (19.5 to 243.0)
**Concentration at T2, mg/L**	74.5 (52.0 to 75.0)	21.6 (7.8 to 80.2)	89.5 (55.0 to 211.0)	93.4 (50.5 to 165.0)	22.1 (12.9 to 37.0)	168.4 (49.0 to 395.0)
**Concentration at target, mg/L**	41.6 (34.3 to 58.2)	18.6 (6.1 to 41.0)	82.0 (43.0 to 174.8)	79.3 (9.6 to 202.0)*	16.1 (9.6 to 30.5)	140.0 (42.0 to 349.0)*
**T >4 × tMIC, h**	7.2 (6.2 to 14.0)	7.0 (4.5 to 11.8)	6.3 (1.0 to 7.7)	15.5 (6.8 to 55.8)	7.1 (6.0 to 11.3)	7.7 (6.1 to 12.4)
**Adequate concentrations**	3 (100%)	9 (36%)	1 (14.3%)	1 (16.7%)	9 (47.4%)	3 (23.1%)
**Excessive concentrations**	0 (0%)	15 (60%)	3 (42.9%)	4 (66.7%)	10 (52.6%)	9 (69.2%)
**SOFA score**	11 (4 to 14)	14 (4 to 19)	12 (5 to 16)	12 (9 to 13)	12 (5 to 20)	13 (9 to 22)
**CRRT blood flow, mL/minute**	140 (130 to 150)	150 (130 to 200)	150 (140 to 180)	140 (100 to 200)	150 (120 to 300)	150 (100 to 180)
**Dialysate, mL/h**	0 (0 to 1,500)	1,500 (0 to 2,000)	1,500 (0 to 2,000)	0 (0 to 1,500)	1,000 (0 to 2,000)	0 (0 to 2,000)
**Ultrafiltrate, mL/h**	1,000 (1,000 to 1,750)	2,000 (100 to 2,000)	1,500 (1,500 to 2,000)	1,500 (1,000 to 3,000)	1,500 (100 to 2,000)	2,000 (1,500 to 2,000)
**CRRT intensity, mL/kg/h**	16.6 (10.5 to 47.7)	36.14 (8.9 to 57.14)	31.5 (15.7 to 57.5)	24.0 (16.6 to 50)	34.2 (16.6 to 85.1)	28.6 (21.4 to 61.5)

The results are presented as median (25th to 75th percentiles for time to sampling) and ranges for all pharmacodynamics and continuous renal replacement therapy (CRRT) data.

*P <0.05. TDM, therapeutic drug monitoring; CEF, ceftazidime or cefepime; MEM, meropenem; TZP, piperacillin/tazobactam; SOFA, sepsis organ failure assessment; t_1/2_, half-life time; T0, trough concentration; T2, concentration 2 h after the onset of drug administration; T >4 × tMIC, time above four times the target minimum inhibitory concentration for *P. aeruginosa*.

**Figure 1:**
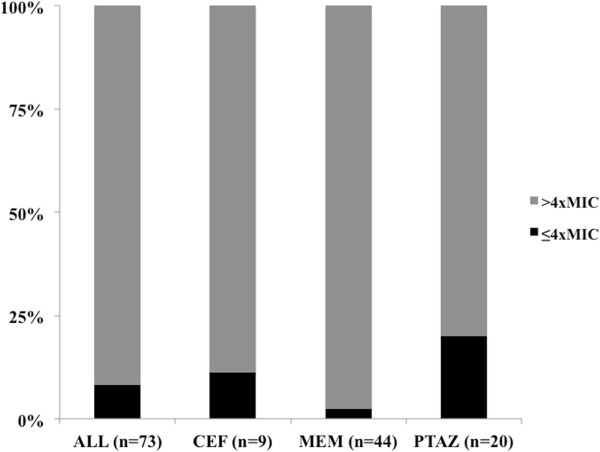
**Proportion of patients with drug concentrations below or above four times the minimal inhibitory concentration (MIC) of *Pseudomonas aeruginosa*, for the different antibiotics.** CEF, cephalosporins; MEM, meropenem; TZP, piperacillin/tazobactam.

**Figure 2:**
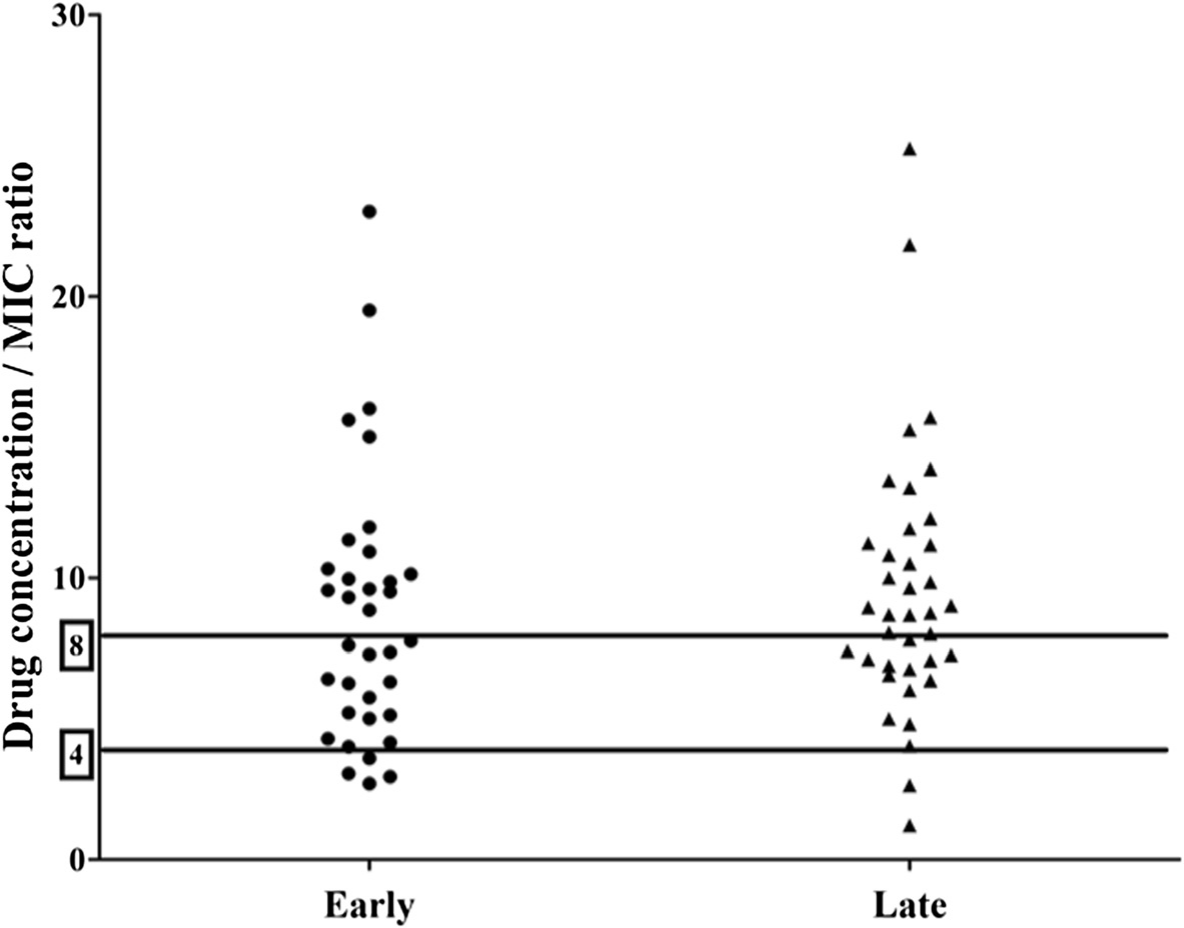
**Distribution of the ratio between drug concentrations and the minimal inhibitory concentration (C/MIC) of *Pseudomonas aeruginosa*.** Drug concentrations were considered at 40%, 50% and 70% for meropenem, piperacillin/tazobactam, and cephalosporin, respectively, and separated according to the early or late phase of therapy. Solid lines indicate a C/MIC of 4 and 8.

Target concentrations were reached in 67/73 (92%) of the TDMs (Figure 1); specifically, 8/9 TDMs for CEF (89%), 43/44 (98%) for MEM and 16/20 (80%) for TZP. The proportions of patients reaching target drug concentrations were similar in the ET and LT phases (Table 3). The % T >4 × tMIC was highly variable among patients (Figure 2) and drug concentrations were excessive in 39 (53%) of the TDMs, 18/35 (51%) in the ET and 22/38 (58%) in the LT phase (Table 3). Calculating the proportion of TDMs with insufficient % T >4 × MIC for several MICs, we found no insufficient drug concentrations for CEF with MIC of 2 mg/L or less, for TZP with MIC of 8 mg/L or less and MEM for MIC of 1 mg/L or less (Table 4).

**Table 4:** Proportion of therapeutic drug monitoring (TDMs) with insufficient % time (T) >4 × the minimal inhibitory concentration (MIC) for various MICs

		**Insufficient drug concentrations**		
**MIC (mg/L)**	**Target concentrations (mg/L)**	**CEF (n = 9)**	**TZP (n = 20)**	**MEM (n = 44)**
**32**	**128**	8 (89)	9 (45)	44 (100)
**16**	**64**	1 (11)	**4 (20)**	44 (100)
**8**	**32**	**1 (11)**	0	41 (93)
**4**	**16**	1 (11)	0	20 (45)
**2**	**8**	0	0	**1 (2)**
**1**	**4**	0	0	0
**0.5**	**2**	0	0	0

Data are expressed as counts (percentage). Boldface values indicate MIC corresponding to European Committee on Antimicrobial Susceptibility Testing (EUCAST) clinical breakpoints for *Pseudomonas aeruginosa*. CEF, cephalosporins; TZP, piperacillin/tazobactam; MEM, meropenem.

**Figure 3:**
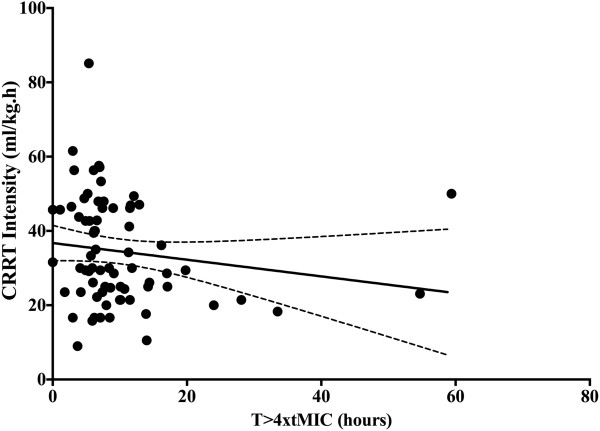
**Correlation between continuous renal replacement therapy (CRRT) intensity and the time (h) above four times the minimal inhibitory concentration (MIC) of *Pseudomonas aeruginosa*.**

**Figure 4:**
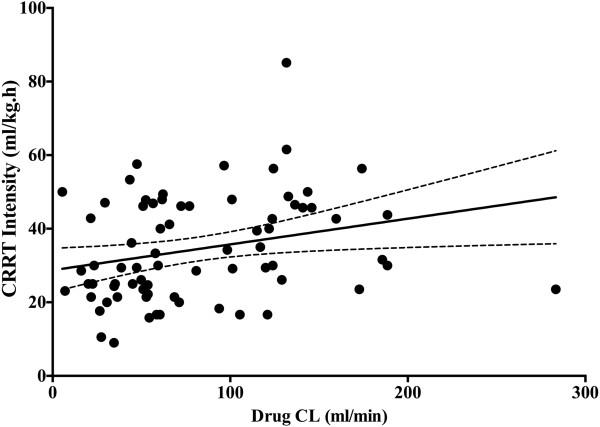
Correlation between continuous renal replacement therapy (CRRT) intensity and drug clearance (CL).

**Figure 5:**
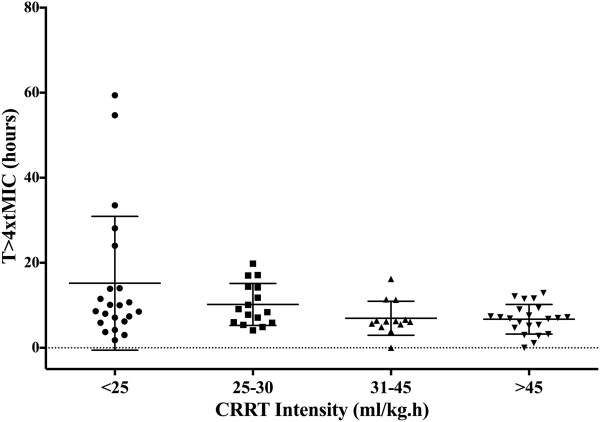
**The time (T) above four times the target (t) minimal inhibitory concentration (MIC) (T > × of *Pseudomonas aeruginosa *was lower in patients with higher continuous renal replacement therapy (CRRT) intensity (<25 mL/kg.h = 9.3 (ranges = 1.8 to 59.4) h; 25 to 30 mL/kg.h = 8.7 (4.1 to 19.8) h; 31 to 45 mL/kg.h = 6.2 (0 to 16.2) h; >45 mL/kg.h = 6.9 (0 to 12.9) h; *P*** **= 0.01).**

**Figure 6:**
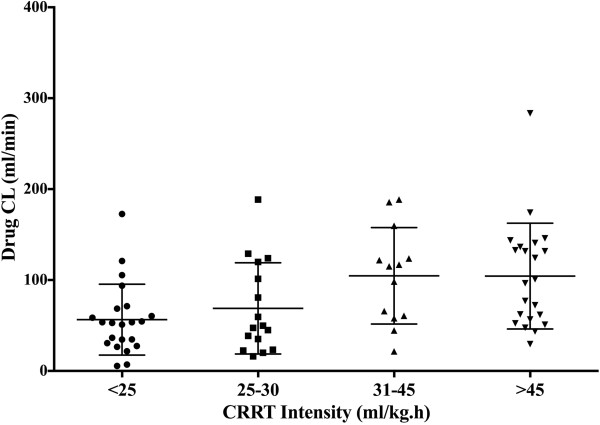
**Drug clearance (CL) was greater in patients with higher continuous renal replacement therapy (CRRT) intensity (<25 mL/kg.h = 53 (ranges = 5 to 172) mL/minute; 25 to 30 mL/kg.h = 49 (16 to 188) mL/minute; 31 to 45 mL/kg.h = 115 (21 to 188) mL/minute; >45 mL/kg.h = 99 (30 to 283) mL/minute; *P*** **= 0.02).**

The T >4 × tMIC was inversely correlated with the CRRT intensity (*r* = −0.24, *P* = 0.03) (Figure 3). β-lactam antibiotics CL was also significantly correlated with the CRRT intensity (*r* = 0.32, *P* = 0.007) (Figure 4). Similarly, drug CL was significantly greater in the upper quartile levels of CRRT intensity whereas T >4 × tMIC was significantly lower (Figure 5 and 6). There were no other correlations between PK/PD parameters and clinical, biologic, laboratory or therapeutic variables (including APACHE II score on admission, SOFA score, lactate and albumin levels, arterial partial pressure of oxygen/inspired oxygen fraction (PaO_2_/FiO_2_), mechanical ventilation and vasopressor therapy on the day of TDM).

## Discussion

In this study, we showed that, during CRRT, use of β-lactam antibiotics at doses similar to those used in patients without AKI resulted in drug concentrations above the minimal target threshold in more than 90% of patients, both in the ET and LT phases of therapy. However, there was wide variability in drug concentrations over time, with very high levels in some patients. Significant, although weak, correlations were found between CRRT intensity and T >4 × tMIC and total drug CL; hence, CRRT intensity should be considered when determining dosage strategy in these patients.

During treatment with CRRT, the prediction of β-lactam antibiotics concentrations is challenging, as both Vd and total drug CL may be affected by the type of membrane, the mechanism of epuration (diffusion versus convection), the total delivered dose and the CRRT intensity [21]. Previous studies that have evaluated β-lactam antibiotics concentrations during CRRT have reported conflicting results. In two studies, an MEM regimen of 0.5 to 1.0 g q12h resulted in adequate drug concentrations to treat the GNB that were identified [22,23]. However, for TZP, doses of 4.0 g q12h resulted in insufficient drug levels to treat *Enterobacteriaceae* and *P. aeruginosa*[24], and increasing the drug dose to 4 g q8h maintained TZP concentrations largely above the target threshold of efficacy [17]. Finally, Malone *et al*. [18] showed that although a cefepime regimen of 2 g daily was sufficient to cover susceptible GNB, higher doses of up to 4 g/day were necessary for bacteria with MICs of at least 8 mg/L. Similarly, Matzke *et al*. proposed that usual doses of ceftazidime be administered to maintain drug levels above the target threshold for pathogens with high MICs [11].

On the basis of these findings, recent recommendations propose daily doses of 1 g q12h for MEM, 2 g q12h for CEF and 4 g q8h for TZP [10], with minor alterations if CVVH or CVVHDF techniques are used. However, these studies were limited by small patient-cohorts, by the use of different CRRT devices and techniques, by the analysis of stable predefined CRRT settings and by the evaluation of various MIC targets. Hence, these data are difficult to generalize to larger ICU populations, may not be relevant if CRRT settings are modified over time, which is common practice, and may not provide an adequate daily dose for the treatment of less susceptible GNB. Indeed, Seyler *et al*. showed that these recommended doses resulted in adequate β-lactam antibiotics concentrations only for pathogens with low MICs and that increased drug doses should be considered in the treatment of less susceptible strains [15]. However, although such an approach may limit under-dosing if *P. aeruginosa* or other less susceptible strains are implicated, for more susceptible pathogens, drug regimens may need to be reduced. Moreover, more than 50% of samples in our study revealed very high drug concentrations in both the ET and LT phases. Drug accumulation and excessive β-lactam antibiotic concentrations may lead to adverse events, including neurological toxicity. Hallucinations, confusion, and seizures have been reported as a consequence of high β-lactam antibiotic concentrations, mostly in patients with renal impairment [25,26], but also in patients with normal kidney function [27]. The mechanism of cerebral toxicity seems to be related to the drug interaction with the GABA-A receptor and is concentration-dependent [28]. Smith *et al*. reported a case of cefepime-related seizures during CRRT in a septic shock patient with a plasma concentration of 73.8 mg/L, but cerebrospinal fluid levels of 6.1 mg/L; seizures resolved after drug discontinuation [29]. Chapuis *et al*. described two patients who developed seizures with trough cefepime concentrations >20 mg/L [30]. We did not collect neurological data in our patients so we cannot relate drug levels to neurological complications.

With the high PK variability among patients, an important finding of our study is the correlation between CRRT intensity and drug CL. In a recent study, Roberts *et al*. found no association between broad-spectrum β-lactam antibiotics and PK and CRRT flow rate [14]. However, the sample size was very limited and drug regimens differed among patients. In other studies, the total CL of TZP, MEM and CEF were strongly correlated to dialysate and ultrafiltration rates [11,13,24]. In a study in six patients, Valtonen *et al*. showed that MEM CL was significantly lower during a flow rate of 1 L/h using CVVH when compared to 2 L/h using CVVHDF [13]. In another study, the same authors showed that TZP CL increased from 3.89 ± 1.23 L/h to 5.48 ± 2.11 L/h when CRRT intensity was doubled [24]. More recently, Covajes *et al*. reported that high CRRT intensity was independently associated with lower vancomycin levels on day 1 of therapy [31]. Although other factors, such as filter age, membrane absorption and residual renal function are all potential confounders when trying to determine a clear relationship between β-lactam antibiotic elimination and CRRT intensity, this parameter should be taken into account and prospectively studied when evaluating optimal dosing strategy during CRRT.

Our study has some limitations. First, β-lactam antibiotics concentrations were sampled at different time points after the onset of therapy and this may not always represent a steady state. Second, because of the retrospective nature of the study, it was not possible to correct drug prescription for delay and interruption of CRRT during therapy, which may have altered drug CL. In addition, unbound free drug concentrations are a major determinant of the total antibiotic CL. Drug binding to circulating proteins, such as albumin, contributes to the decrease in the passage of TZP across CRRT membrane, although it cannot completely explain the findings obtained in several clinical studies [32]. Although we considered that protein binding was negligible for CEF and MEM, we did not measure free drug levels for TZP, which has an estimated protein binding of 25 to 30%, and this may be a significant confounder in this setting. Third, our target concentrations could be criticized because no clear threshold of effectiveness has been defined for β-lactam antibiotics in the treatment of life-threatening infections, and T > MIC and T >4 × MIC have both been shown to be associated with a better clinical response [33,34]. Significant variation observed in the PK targets supports the need for further studies to define optimal drug concentrations for ICU patients [35]. Also, we did not specifically measure MICs of isolated bacteria and significant differences in final results would have been obtained if target MICs other than those of *P. aeruginosa* were considered. Indeed, when the isolated bacteria are found to be susceptible and have a low MIC (as for some *Enterobacteriaceae*), it is hard to justify maintaining the same therapeutic targets as for *Pseudomonas*, and exposing the patients to high concentrations and to potential toxicity. Accordingly, dose adjustment should probably be considered along with drug de-escalation, not only according to TDM but also to bacteria susceptibility and MIC [36,37]. Fourth, no data on efficacy that is, clinical or microbiological responses) were collected so that no conclusion on the role of routine measurements of β-lactam antibiotics concentration can be drawn from our results. Fifth, although we compared drug concentrations in the early and late phase of therapy, we did not assess the two time points for every patient and this latter approach would have probably been more informative in this setting. Finally, we did not collect the number of patients treated with CRRT and receiving these drugs but whose β-lactam concentrations were not assessed; this may be an important selection bias with only the most severe patients who were included, which may lead to an overestimation of the problem.

## Conclusions

During CRRT, β-lactam antibiotics regimens similar to those recommended for patients with normal renal function should be given to avoid under-dosing as empirical therapy. However, drug accumulation occurs rapidly and daily doses should be rapidly reduced, especially in case of very susceptible bacteria. Given the wide variability in drug PK parameters in this population of patients, TDM could be considered to adjust drug regimens. Drug prescription should also take into account the intensity of CRRT.

## Key messages

• The use of standard doses of β-lactam antibiotics during CRRT may result in inadequate serum concentrations to treat less susceptible Gram-negative strains, such as *P. aeruginosa*.

• During CRRT, the administration of drug regimens similar to those patients with normal renal function reduced the proportion of inadequate serum concentrations to 10%.

• Nevertheless, 53% of samples were associated with very high drug levels and daily drug regimens may need to be adapted accordingly to avoid adverse events.

## Abbreviations

AKI: acute kidney injury; APACHE: acute physiology and chronic health evaluation; CEFP: ceftazidime or cefepime; CL: clearance; CrCl: creatinine clearance; CRRT: continuous renal replacement therapy; CVVHDF: continuous veno-venous hemodiafiltration; CVVHF: continuous veno-venous hemofiltration; ECMO: extracorporeal membrane oxygenation; ET: early phase; EUCAST: European Committee on Antimicrobial Susceptibility Testing; GNB: Gram-negative bacteria; LT: late phase; MEM: meropenem; MIC: minimal inhibitory concentration; PaO_2_/FiO_2_: arterial partial pressure of oxygen/inspired oxygen fraction; PK: pharmacokinetics; SOFA: sequential organ failure assessment; TDM: therapeutic drug monitoring; TZP: piperaacillin/tazobactam; Vd: volume of distribution.

## Competing interests

The author declared that they have no competing interests.

## Author contributions

MB, FJ and FST contributed to the conception and design of the study protocol. GSC, LS, JLV and MH participated in the coordination of the study and data collection. FC performed the pharmacokinetics analyses and contributed to the analysis and interpretation of the data. MB, GCS, FJ, JLV and FST drafted the manuscript. All authors were involved in revising it critically for important intellectual content. All authors read and gave final approval of the present version of the manuscript to be published.
